# Characterization of the role of putative *Aeromonas caviae*-specific virulence factor, *flgB,* in virulence and host–pathogen interactions

**DOI:** 10.1128/jb.00339-25

**Published:** 2025-12-29

**Authors:** Bernadette A. Hritzo, Jane M. Michalski, David A. Rasko

**Affiliations:** 1 Institute for Genome Sciences, University of Maryland School of Medicine, Baltimore, Maryland, USA; 2 Department of Microbiology and Immunology, University of Maryland School of Medicine 12264, Baltimore, Maryland, USA; 3 Department of Microbial Pathogenesis, University of Maryland School of Dentistry 89015, Baltimore, Maryland, USA; University of Virginia School of Medicine, Charlottesville, Virginia, USA

**Keywords:** *Aeromonas*, *Aeromonas caviae*, emerging pathogen, comparative genomics, virulence factor, host–pathogen interactions

## Abstract

*Aeromonas caviae,* Gram-negative bacteria ubiquitous in the environment, are an emerging human pathogen associated with various infectious diseases, particularly gastroenteritis. Despite recent studies demonstrating *A. caviae* is the most predominant *Aeromonas* species underlying human infection, *A. caviae* remains understudied, and no *A. caviae*-specific virulence factors associated with human disease have been identified. To identify *A. caviae*-specific putative virulence factors, we conducted comparative genomic analyses among clinical *Aeromonas* isolates (*n* = 431), which identified a variant of *flgB,* predicted to encode a polar flagellum machinery protein, as over-represented in *A. caviae* isolates. To examine the role of *flgB* in virulence and host–pathogen interactions, we generated an *A. caviae flgB* deletion mutant and genetic complementation constructs. Swimming motility and polar flagella assembly were abolished in the mutant and functionally rescued with genetic complementation. As it remains unknown where *A. caviae* infects the human gastrointestinal tract, we assessed host–pathogen interactions in HT-29 and Caco2 human intestinal cell lines, representative of the large and small intestine, respectively. Deletion of *flgB* significantly decreased bacterial adherence in only HT-29 cells and also decreased production of proinflammatory cytokines, IL-8, IL-13, IL-1β, and IL-6, by both cell types. Given the lack of relevant mammalian models for studying most enteric pathogens *in vivo,* we characterized *in vivo* virulence in a *Galleria mellonella* larval survival model, where the *flgB* deletion modestly attenuated virulence. Deletion of *flgB* altered aspects of virulence and host–pathogen interactions, and this study provides a framework for identification and characterization of *A. caviae*-specific putative virulence factors.

**IMPORTANCE:**

*Aeromonas caviae* is an emerging human bacterial pathogen associated with gastroenteritis, wound infections, and numerous other infectious diseases. Recent studies demonstrate that *A. caviae* accounts for the greatest burden of human *Aeromonas* infections. Despite this, *A. caviae* is understudied as a human pathogen. To address this gap in knowledge, this study characterizes *A. caviae-*specific virulence genes. We examined 431 clinical *Aeromonas* isolates using comparative genomics and identified and functionally characterized a putative *A*. caviae-specific virulence factor*, flgB*. Genetic deletion of *flgB* in *A. caviae* resulted in deficiencies in bacterial motility, adherence, host-cell proinflammatory cytokine production, and *in vivo* virulence in an invertebrate model. This work establishes the foundation for further study of additional *A. caviae-*specific virulence factors.

## INTRODUCTION

*Aeromonas* spp., Gram-negative bacteria commonly present in the environment, are emerging human pathogens associated with various infectious diseases in both immunocompetent and immunocompromised humans (1–15). Despite the increasing evidence of *Aeromonas’* association with human infection and disease, *Aeromonas* spp. have only recently been recognized and confirmed as independent human pathogens (4, 6–10). Historical ambiguity in defining *Aeromonas* spp. as human pathogens (10, 16, 17) has resulted in a significant gap in knowledge regarding *Aeromonas* virulence and pathogenesis in humans. This ambiguity stemmed in part from the lack of routine speciation of *Aeromonas* isolates in clinical settings and surveillance studies, as *Aeromonas* cannot be reliably speciated with 16S-rRNA sequencing or standard microbiological and biochemical assays (6, 16, 18–21). This lack of species-level resolution precludes the ability to more comprehensively assess which species are contributing to human infection and virulence across species. This highlights the need to understand both the species relevant to human infection and *Aeromonas* species-specific virulence and pathogenesis in the context of human infection.

In the Global Enteric Multicenter Study (GEMS) (4, 11), a large case-control study surveying the burden of pathogens in pediatric diarrheal disease in Sub-Saharan Africa and Asia, *Aeromonas* spp. was among the top six enteric pathogens most commonly isolated from diarrheal cases in each age group examined and was isolated in >22% of diarrheal cases in Pakistan and Bangladesh (4). However, GEMS *Aeromonas* isolates were not speciated in real-time. Further studies by our group utilized genome sequencing and phylogenetic comparison to speciate a subset of GEMS *Aeromonas* spp. isolates (*n* = 431) (22). These analyses identified *Aeromonas caviae* as both the most prevalent species isolated, accounting for >50% of all isolates, and the only species significantly associated with human pediatric diarrheal disease (22). A recent study by Fernández-Bravo and Figueras identified that ~95% of human *Aeromonas* infections are attributed to only four *Aeromonas* species, *A. caviae*, *A. dhakensis, A. veronii,* and *A. hydrophila,* and *A. caviae* was most prevalent, accounting for 37.3% of infections (6). Augmented by the enhanced resolution of whole-genome sequencing and molecular methods, additional studies have recently established the prevalence of *A. caviae* in human *Aeromonas* infections (5, 23). In a multicenter cohort study of *Aeromonas* infections in adults in Japan, hepatobiliary disease was the most common *Aeromonas-*associated pathology, and *A. caviae* was the most abundant species, with isolation in 60% of cases (87/144) (5). Additionally, in a non-age restricted study of diarrheal cases in Northern Italy, *Aeromonas* was the second leading cause of bacterial diarrheal infection, accounting for 24.6% of cases, and *A. caviae* was isolated from 75% of the *Aeromonas* diarrheal cases (30/40) (23). Taken together, these studies form burgeoning evidence that *A. caviae* is a significant human pathogen and demonstrate the significant burden of *A. caviae* in human *Aeromonas* infections.

Despite these findings, and other evidence of *A. caviae* pathogenicity (15, 24) and burden in human *Aeromonas* infections (2), the pathogenicity of *A. caviae* has been debated (15, 25, 26). *A. caviae* are known to lack aerolysin, a T3SS, and heat-stable enterotoxin (*ast*), which are virulence factors commonly present in the other clinically relevant human-associated *Aeromonas* species (12, 25–29). The lack of these virulence factors resulted in the assumption that *A. caviae* was non-pathogenic (26). These assumptions have resulted in a systematic lack of understanding of *A. caviae* virulence in the human host, highlighting the urgent need for identification and characterization of *A. caviae* virulence factors.

In this study, we utilized comparative genomic analyses in >400 human-associated clinical GEMS *Aeromonas* spp. isolates (22) to identify genomic features specific to *A. caviae* with a putative role in virulence. We selected one representative putative *A. caviae*-specific virulence factor, *flgB,* predicted to encode a rod protein of the polar flagellar machinery and assembly apparatus (30–33), to functionally characterize in virulence and host–pathogen interactions. Given the role of *A. caviae* polar flagella in motility and adherence to human cell lines in limited previous studies (34–39), we hypothesized that deletion of *flgB* would affect virulence and host–pathogen interactions. To test our hypothesis, we genetically deleted *flgB* in a representative GEMS *A. caviae* isolate. Deletion of *flgB* abolished swimming motility and polar flagella assembly, decreased bacterial adherence to and proinflammatory cytokine production by human intestinal epithelial cell lines, as well as modestly increased survival in a *Galleria mellonella* larval *in vivo* model. These studies highlight the ability of this framework to identify and characterize *A. caviae*-specific genomic features that impact virulence and host–pathogen interactions.

## RESULTS

### Identification and mutagenesis of *A. caviae*-specific putative virulence factor, *flgB*

Mounting evidence of *A. caviae*’s prevalence in human infection (5, 6, 23, 28, 40) indicates *A. caviae* virulence, yet the virulence factors of human-associated *A. caviae* are not well characterized (27, 29). Previously, we conducted genome sequencing and phylogenetic comparison to speciate a representative subset of clinical human-associated *Aeromonas* isolates (*n* = 431) from GEMS (4, 11, 22). This analysis identified eight species: *A. caviae* (*n* = 230)*, A. veronii* (*n* = 124)*, A. enteropelogenes* (*n* = 29), *A. dhakensis* (*n* = 21), *A. jandaei* (*n* = 14), *A. taiwanensis* (*n* = 8), *A. sanarellii* (*n* = 4), and *A. hydrophila* (*n* = 1). Notably, *A. caviae* was the most prevalent species isolated (53.4%, 230/431) and the only species significantly associated with diarrheal disease (*P* = 0.004) compared to non-diarrheal controls (22).

We conducted comparative genomic analyses among these 431 *Aeromonas* genomes, utilizing the Roary (41) and Scoary (42) pipelines to compare *A. caviae* genomes (*n* = 230) to the genomes of the seven other *Aeromonas* species (*n* = 201). These analyses identified >300 genes that were significantly more abundant in *A. caviae* isolates than in the isolates of the other seven species (*P* < 0.0005). From these genes, we identified putative *A. caviae*-specific virulence factors as genes present in ≥90% of *A. caviae* isolates and ≤10% of other *Aeromonas* species isolates predicted to encode virulence factors in other enteric pathogens, such as genes involved in bacterial motility and adherence (8, 31, 34, 35). One such putative virulence gene was an *flgB* gene variant, which was significantly over-represented in *A. caviae* isolates (229/230 [99.6%]) compared to the isolates of the other seven *Aeromonas* species (12/201 [5.9%]) (*P* < 0.0005). Phylogenetic comparison of *flgB* nucleotide sequence across the 431 isolates confirmed the presence of an *flgB* gene in all *Aeromonas* species isolates. This analysis also confirmed the species specificity of *flgB* (Fig. S1A). The inferred *flgB* phylogenetic tree separates each species into distinct clades, with the exception being the *flgB* of *A. taiwanensis* and *A. sanarellii* isolates, which group with a subset of *A. caviae* isolates in a sub-clade (dark blue) nested within the *A. caviae* clade (light blue) (Fig. S1A). Allelic variation of *flgB* nucleotide sequence for species-specific variants (aligned to the *flgB* of a representative *A. caviae*) was observed as follows: *A. caviae* (7.9%)*, A. sanarellii* (7.9%)*, A. taiwanensis* (8.9%), *A. hydrophila* (15.1%), *A. dhakensis* (15.3%), *A. veronii* (22.5%), *A. jandaei* (19.3%), and *A. enteropelogenes* (19.5%) (Fig. S1B). Comparison of amino acid identity of the *flgB* from all *Aeromonas* species (aligned to the *flgB* of a representative *A. caviae*) ranged as follows: *A. caviae* (95.5%–100%), *A. sanarellii* (95.5%), *A. taiwanensis* (95.5%–96.2%), *A. hydrophila* (92.4%), *A. dhakensis* (90.9%–91.7%), *A. veronii* (88.6%–90.2%), *A. jandaei* (89.5%–90.2%), and *A. enteropelogenes* (90.2%–90.9%) (Fig. S1B). Amino acid similarity across apparent species-specific *flgB* variants ranged as follows: *A. caviae* (97%–100%), *A. sanarellii* (97%), *A. taiwanensis* (97%–98%), *A. hydrophila* (97%), *A. dhakensis* (95%–96%), *A. veronii* (93%–95%), *A. jandaei* (95%), and *A. enteropelogenes* (95%) (Fig. S1B). Nucleotide and amino acid sequence alignments for representative species-specific *flgB* variants are presented in Fig. S1C and D, respectively.

The *flgB* was predicted to encode a rod protein of the polar flagella basal body (30, 31), a component of the machinery involved in polar flagella assembly (32, 33). As *A. caviae* polar flagella (37) have been shown to confer swimming motility (34–36) and mediate adherence to some examined human cell lines (36–39), we hypothesized a putative role for *flgB* in *A. caviae* virulence. We selected this *A. caviae-*specific *flgB* as a representative putative virulence factor for mutagenesis and characterization in virulence and host–pathogen interactions, as there are functional assays with defined phenotypes to assess flagella functionally.

To assess the role of *flgB* in bacterial virulence and host–pathogen interactions, we generated an isogenic *flgB* deletion mutant (designated Δ*flgB*) in a representative human-associated *A. caviae* isolate from GEMS, 600239, utilizing the Lambda Red system (43, 44) (Table 1). We also generated two plasmid expression constructs for in *trans* complementation of our deletion mutant (Table 1). In the first construct (pBAH001), *flgB* is expressed from the *Escherichia coli ompC* promoter (P*_ompC_*), which can be modulated by the osmolarity of the culture conditions (45, 46). To attempt to replicate endogenous regulation and *flgB* expression from the native promoter, the second construct (pBAH002) was engineered to contain *flgB* and 500 base pairs of sequence upstream of *flgB*, presumably containing the native promoter. Whole-genome sequencing confirmed definitive *flgB* deletion, with no other genetic effects, and successful introduction of complementation constructs in respective strains [designated Δ*flgB* (pBAH001) and Δ*flgB* (pBAH002)]. Bacterial fitness was not affected by *flgB* deletion nor complementation with either construct as compared to wild-type 600239, as determined by conducting growth curves in respective nutrient-rich media (Fig. S2).

**TABLE 1:** Strains and plasmids used in this study

ID	Description	Reference/source
Strains		
600239	Wild-type *A. caviae* clinical isolate; isolated from human (pediatric) diarrheal stool sample (Bangladesh)	(11)
Δ*flgB*	Stable chromosomal deletion of *flgB* in 600239	This study
Δ*flgB* (pBAH001)	Δ*flgB* complemented in *trans* with pBAH001 (Kan/Neo^R^, 50 µg/mL); *flgB* expression from osmoregulated *E. coli ompC* promoter (P_*ompC*_)	This study
Δ*flgB* (pBAH002)	Δ*flgB* complemented in *trans* with pBAH002 (Kan/Neo^R^, 50 µg/mL); *flgB* expression from native promoter	This study
Plasmids		
pKM200	Lambda Red recombinase (Cm^R^, 20 µg/mL); temperature-sensitive origin of replication (30°C)	(44)
pKD4	Helper plasmid containing Kanamycin/Neomycin cassette (Kan/Neo^R^, 50 µg/mL)	(43)
pCP20	Plasmid containing flippase (Cm^R^, 20 µg/mL); temperature-sensitive origin of replication (30°C)	(43)
pCR-Blunt II-TOPO::*flgB*	Invitrogen pCR-Blunt II-TOPO::600239*flgB* (Kan/Neo^R^, 50 µg/mL)	This study
pCR-Blunt II-TOPO::*flgB*+500	Invitrogen pCR-Blunt II-TOPO::600239*flgB*+500 bp upstream sequence (Kan/Neo^R^, 50 µg/mL)	This study
pSEC10	Low copy number vector (5 copies/cell) for complementation construct; contains osmoregulated *E. coli ompC* promoter (P_*ompC*_) (Kan/Neo^R^, 50 µg/mL)	(45, 46)
pSEC10-M	Low copy number vector (five copies/cell) for complementation construct; lacks osmoregulated *E. coli ompC* promoter (P_*ompC*_) (Kan/Neo^R^, 50 µg/mL)	(45–47)
pBAH001	pSEC10::600239*flgB* (Kan/Neo^R^, 50 µg/mL)	This study
pBAH002	pSEC10-M::600239*flgB*+500 bp upstream sequence (Kan/Neo^R^, 50 µg/mL)	This study

### Deletion of *flgB* abrogates aqueous motility and polar flagella production

**Fig 1:**
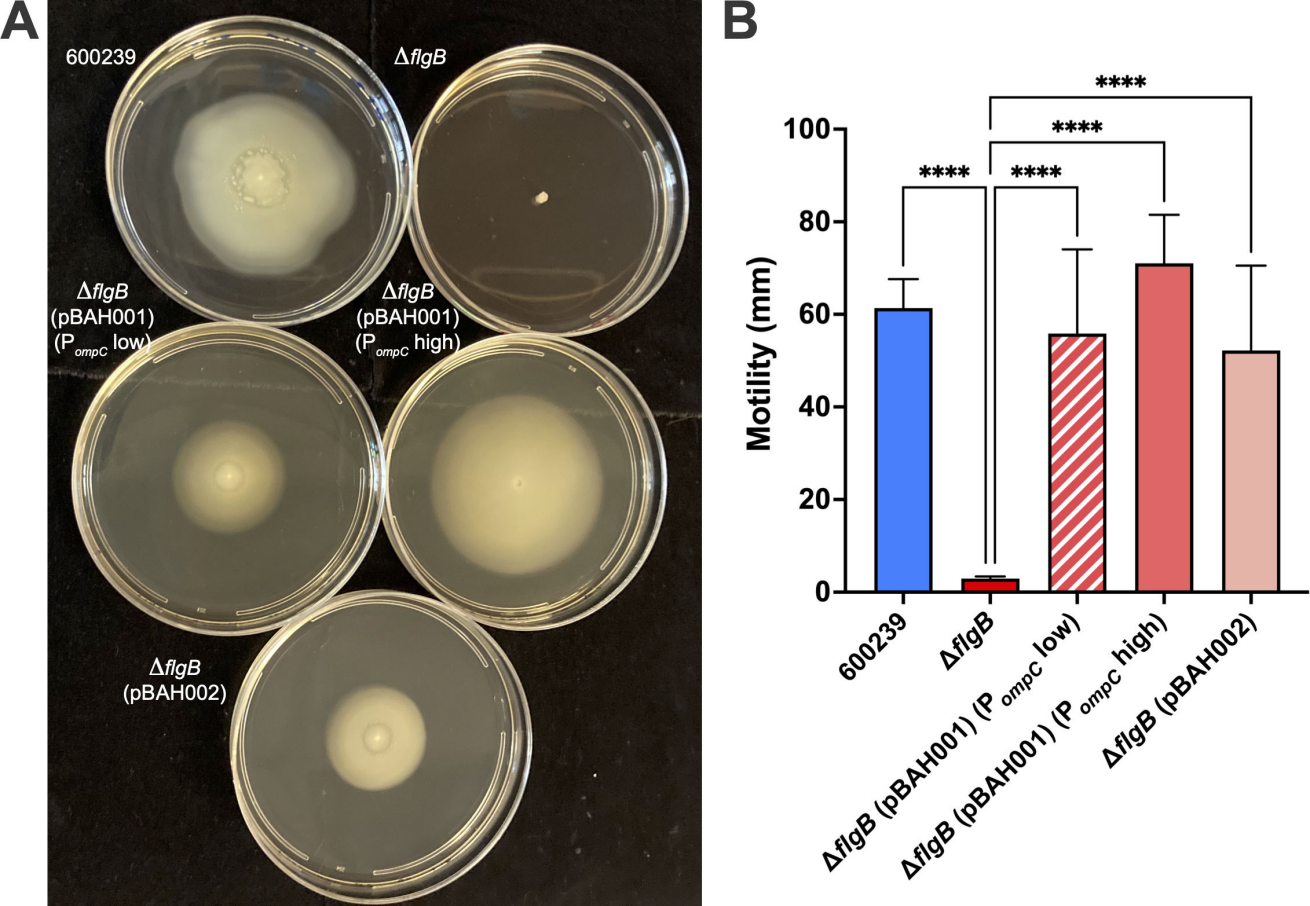
Swimming motility of *A. caviae* strains by soft agar assay. The wild-type 600239, Δ*flgB* mutant, and complemented strains, Δ*flgB* (pBAH001) [*flgB* expression from the osmoregulated *E. coli ompC* promoter (P*_ompC_*)] and Δ*flgB* (pBAH002) (expression from the native promoter), were inoculated into soft agar plates (0.25% agar) and incubated overnight at 37°C. The Δ*flgB* (pBAH001) strain was cultured in media containing 3 g/L NaCl (annotated P*_ompC_* low) or 10 g/L NaCl (annotated P*_ompC_* high) to examine motility when modulating expression from P*_ompC_*. A representative image of the motility of each strain in soft agar plates is shown in panel **A**. Migration from the inoculation point was measured ~22 h post-inoculation (diameter, in mm) (**B**). Groups were compared by ordinary one-way ANOVA with Tukey’s test for multiple comparisons (****, *P* ≤ 0.0001). Data pooled from three independent experiments is shown (**B**).

As *flgB* is predicted to encode a component of the polar flagellar machinery (30–33), we utilized soft agar assays to assess swimming motility. Motility of Δ*flgB* (pBAH001) was assessed when culturing with 3 g/L NaCl (designated P*_ompC_* low) or 10 g/L NaCl (designated P*_ompC_* high), as expression from P*_ompC_* can be modulated by osmolarity (45, 46). A representative image of the motility of all strains in soft agar plates ~22 h post-inoculation is shown in Fig. 1A. The Δ*flgB* mutant exhibited abrogated swimming motility compared to wild-type 600239 (*P* < 0.0001) (Fig. 1B). Motility of Δ*flgB* (pBAH001) (P*_ompC_* low), Δ*flgB* (pBAH001) (P*_ompC_* high), and Δ*flgB* (pBAH002) was restored to wild-type levels (*P* = 0.9430, 0.6920, and 0.7305, respectively) and significantly increased compared to that of Δ*flgB* (*P* < 0.0001, for all) (Fig. 1B), demonstrating functional complementation of swimming motility with both constructs. The motility of Δ*flgB* (pBAH001) (P*_ompC_* low) was not significantly different than that of Δ*flgB* (pBAH001) (P*_ompC_* high) (*P* = 0.4210). Therefore, we conducted all subsequent experiments culturing Δ*flgB* (pBAH001) with 10 g/L NaCl (P*_ompC_* high).

**Fig 2:**
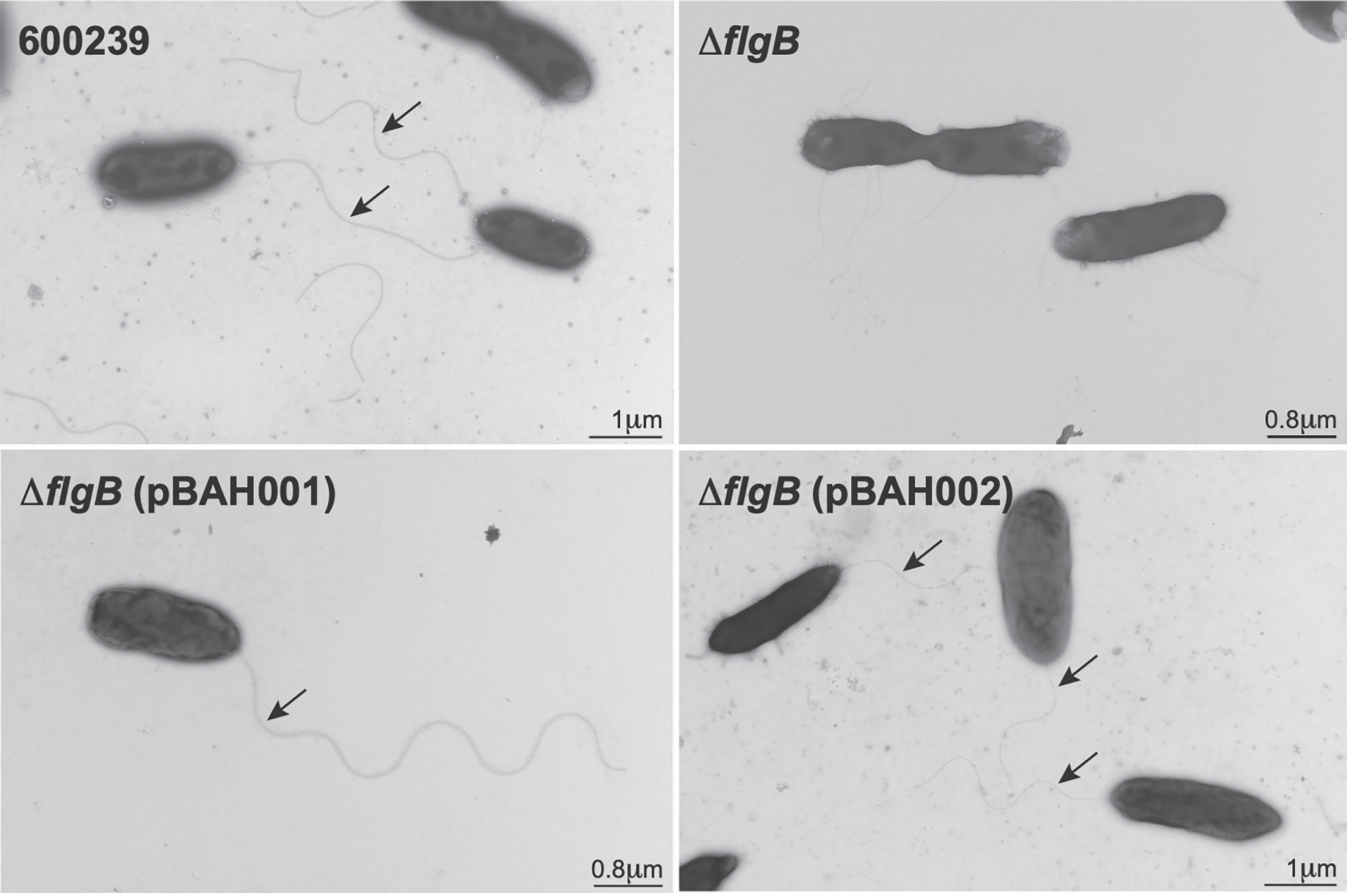
Visualization of polar flagella production in *A. caviae* strains by negative staining and transmission electron microscopy. The wild-type 600239 (upper left), Δ*flgB* mutant (upper right), and complemented strains, Δ*flgB* (pBAH001) (lower left) and Δ*flgB* (pBAH002) (lower right), were mounted on glow-discharged, formvar carbon-stabilized grids, stained with phosphotungstic acid, and visualized with transmission electron microscopy. Representative images are shown. Polar flagella are marked with arrows and scale bars indicate size (in μm).

To examine if the loss of motility was due to defects in polar flagella assembly, we visually assessed polar flagella production in the 600239 (upper left panel), Δ*flgB* (upper right panel), Δ*flgB* (pBAH001) (lower left panel), and Δ*flgB* (pBAH002) (lower right panel) strains using negative staining and transmission electron microscopy (Fig. 2). Polar flagella were present in the 600239 wild type; however, no polar flagella were observed in the Δ*flgB* mutant, indicating deletion of *flgB* abolished polar flagella assembly. Polar flagella were observed in both the Δ*flgB* (pBAH001) and Δ*flgB* (pBAH002) strains, indicating polar flagella assembly was functionally complemented with both constructs.

### Bacterial adherence and proinflammatory cytokine production were altered with *flgB* deletion

Previous studies have demonstrated *A. caviae* polar flagella mediate adherence to non-intestinal human cell lines (36–39, 48); however, there are limited studies assessing *A. caviae* adherence and other host–pathogen interactions in relevant human intestinal cell lines (23, 48–50). Additionally, the location of *A. caviae* binding and infection in the human gastrointestinal tract remains unknown (51). We utilized relevant HT-29 and Caco2 human intestinal epithelial cell lines, representative of colonic cells (52) and small intestinal epithelial cells (38, 52), respectively, to characterize the effects of the *flgB* mutation on bacterial adherence and proinflammatory cytokine production.

**Fig 3:**
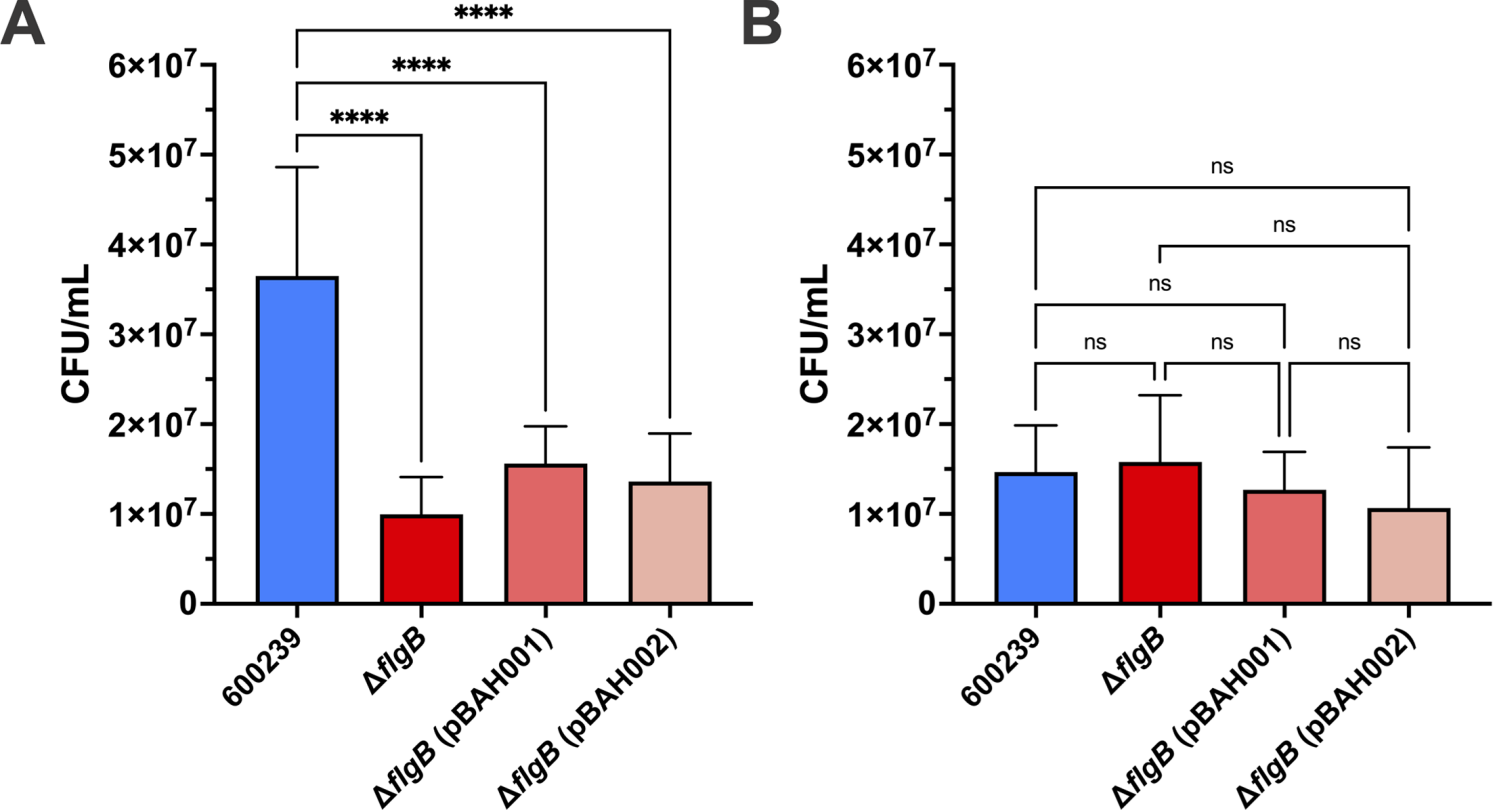
Adherence of *A. caviae* strains in HT-29 and Caco2 intestinal epithelial cell lines. Confluent monolayers of HT-29 (**A**) and Caco2 (**B**) cells were infected with the wild-type 600239, Δ*flgB* mutant, or complemented strains, Δ*flgB* (pBAH001) or Δ*flgB* (pBAH002) (in technical triplicate) at MOI = 0.05. Fresh Dulbecco’s Modified Eagle Medium was used as a negative control. Four hours post-infection, monolayers were washed to remove non-adhered planktonic bacteria, lysed with Triton X, and the resulting mixture was serially diluted and plated for enumeration of CFU of adhered bacteria. Groups were compared by ordinary one-way ANOVA with Tukey’s test for multiple comparisons (****, *P* ≤ 0.0001; ns, non-significant); adherence was not significantly different between any of the experimental groups in the Caco2 cell line (**B**). Data presented is pooled from three independent experiments.

In the HT-29 cells, Δ*flgB* exhibited significantly decreased adherence compared to wild-type 600239 (*P* < 0.0001) (Fig. 3A). Adherence of Δ*flgB* (pBAH001) and Δ*flgB* (pBAH002) was not significantly different compared to that of Δ*flgB* (*P* = 0.3623 and 0.7104, respectively) (Fig. 3A), indicating complementation with either pBAH001 or pBAH002 did not rescue adherence in the HT-29 cells. In the Caco2 cells, *flgB* deletion had no effect on adherence, with similar adherence observed in the wild-type and Δ*flgB* mutant (*P* = 0.9794) (Fig. 3B). Adherence of Δ*flgB* (pBAH001) and Δ*flgB* (pBAH002) was not significantly different than that of the wild-type 600239 (*P* = 0.8981 and 0.5022, respectively), or Δ*flgB* mutant (*P* = 0.7003 and 0.2914, respectively) (Fig. 3B).

**Fig 4:**
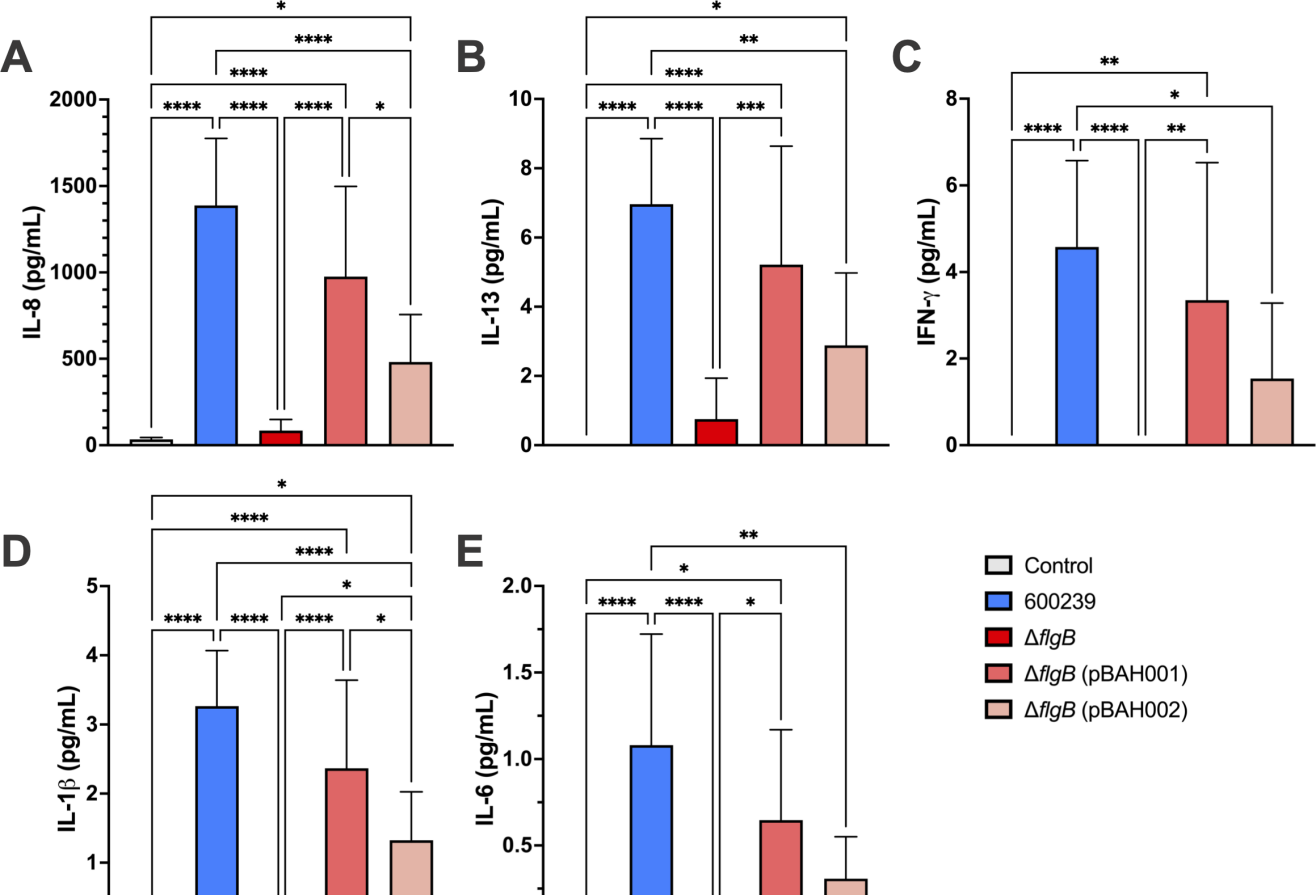
Proinflammatory cytokine production in HT-29 intestinal epithelial cell lines infected with *A. caviae* strains. Confluent monolayers of HT-29 cells were infected with the wild-type 600239, Δ*flgB* mutant, or complemented strains, Δ*flgB* (pBAH001) or Δ*flgB* (pBAH002) at MOI = 0.05 for 4 h. Each strain was assayed in technical triplicate and fresh Dulbecco’s Modified Eagle Medium was used as a negative control. A multiplex cytokine kit was used to measure the production of proinflammatory cytokines, including IL-8 (**A**), IL-13 (**B**), IFN-γ (**C**), IL-1β (**D**), and IL-6 (**E**), in spent media supernatants. Groups were compared by ordinary one-way ANOVA with Tukey’s test for multiple comparisons (****, ***, **, and *, *P* ≤ 0.0001, *P* ≤ 0.001, *P* ≤ 0.01, and *P* ≤ 0.05, respectively). Data pooled from three independent experiments is shown.

**Fig 5:**
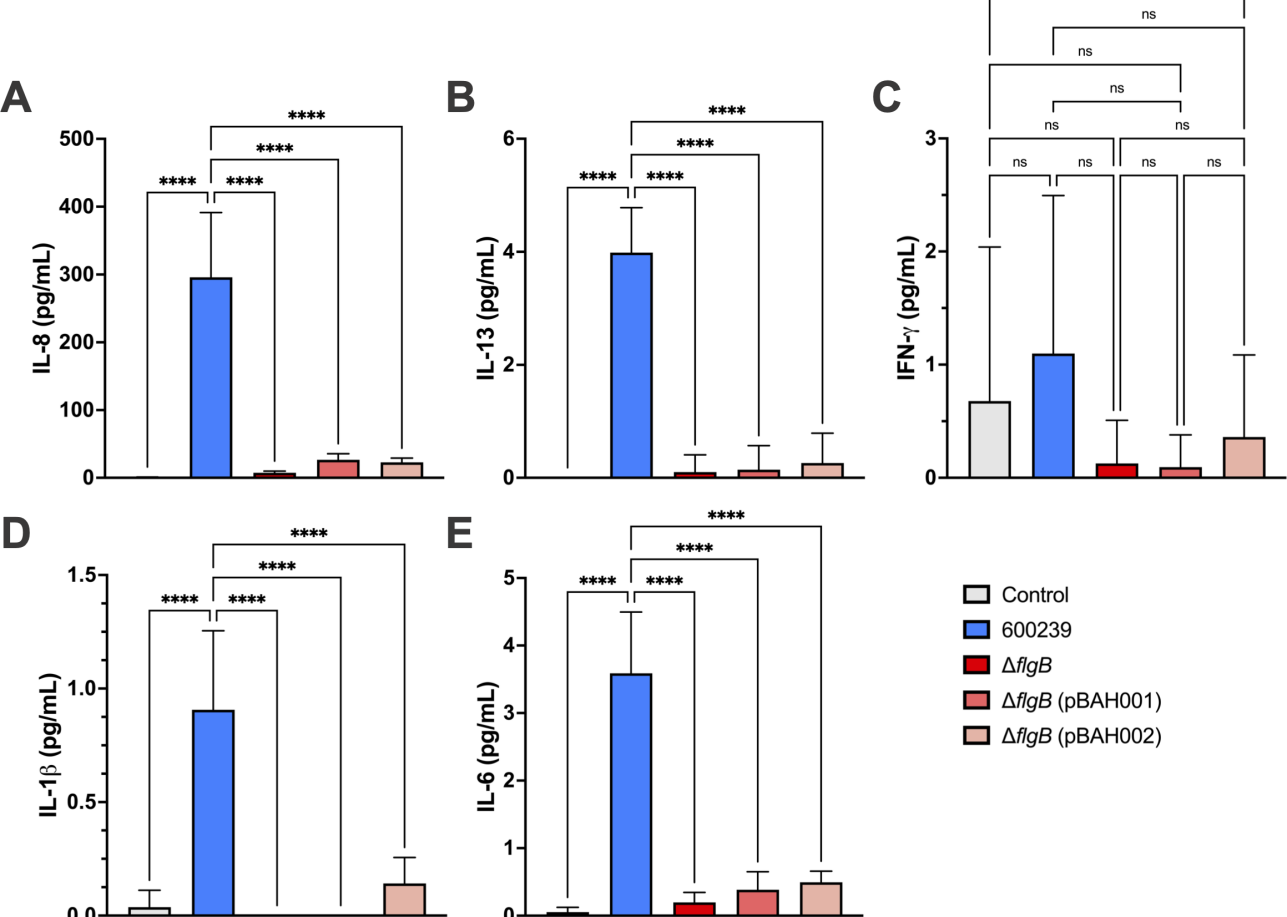
Proinflammatory cytokine production in Caco2 intestinal epithelial cell lines infected with *A. caviae* strains. Confluent monolayers of Caco2 cells were infected with the wild-type 600239, Δ*flgB* mutant, or complemented strains, Δ*flgB* (pBAH001) or Δ*flgB* (pBAH002) at MOI = 0.05 for 4 h. Strains were assayed in technical triplicate and fresh Dulbecco’s Modified Eagle Medium was used as a negative control. A multiplex cytokine kit was used to measure the production of proinflammatory cytokines, including IL-8 (**A**), IL-13 (**B**), IFN-γ (**C**), IL-1β (**D**), and IL-6 (**E**), in spent media supernatants. Groups were compared by ordinary one-way ANOVA with Tukey’s test for multiple comparisons (****, ***, **, and *, *P* ≤ 0.0001, *P* ≤ 0.001, *P* ≤ 0.01, and *P* ≤ 0.05, respectively); (ns, non-significant). Data pooled from three independent experiments is shown.

Production of proinflammatory cytokines interferon-gamma (IFN-γ), tumor necrosis factor-alpha (TNF-α), IL-1β, IL-2, IL-4, IL-6, IL-8, IL-10, IL-12p70, and IL-13 was measured by multiplex cytokine assay using spent media supernatants from the adherence assays in both HT-29 (Fig. 4A through E) and Caco2 (Fig. 5A through E) cells. TNF-α, IL-2, IL-4, IL-10, and IL-12p70 were not produced at 4 h post-infection with any of the bacterial strains, in either HT-29 or Caco2 cell lines (data not shown). Induction of IL-8 (Fig. 4A), IL-13 (Fig. 4B), IFN-γ (Fig. 4C), IL-1β (Fig. 4D), and IL-6 (Fig. 4E) was significantly reduced when infecting HT-29 cells with the Δ*flgB* mutant compared to the 600239 wild type (*P* < 0.0001 for all). Complementation with pBAH001 or pBAH002 partially rescued production of IL-8 (Fig. 4A), IL-13 (Fig. 4B), IFN-γ (Fig. 4C), IL-1β (Fig. 4D), and IL-6 (Fig. 4E), with greater cytokine production occurring with Δ*flgB* (pBAH001) infection compared to Δ*flgB* (pBAH002). IL-8 (Fig. 5A), IL-13 (Fig. 5B), IL-1β (Fig. 5D), and IL-6 (Fig. 5E) production was significantly decreased in Caco2 cells infected with the Δ*flgB* mutant compared to 600239 wild type (*P* < 0.0001 for all). IFN-γ production did not differ significantly between any strains in the Caco2 infections (Fig. 5C). In contrast to observations in the HT-29 cells, there was no restoration of IL-8 (Fig. 5A), IL-13 (Fig. 5B), IL-1β (Fig. 5D), or IL-6 (Fig. 5E) production when infecting Caco2 cells with Δ*flgB* (pBAH001) or Δ*flgB* (pBAH002), indicating a lack of functional complementation with either construct.

### Examination of the role of *flgB* in a *G. mellonella* survival model

**Fig 6:**
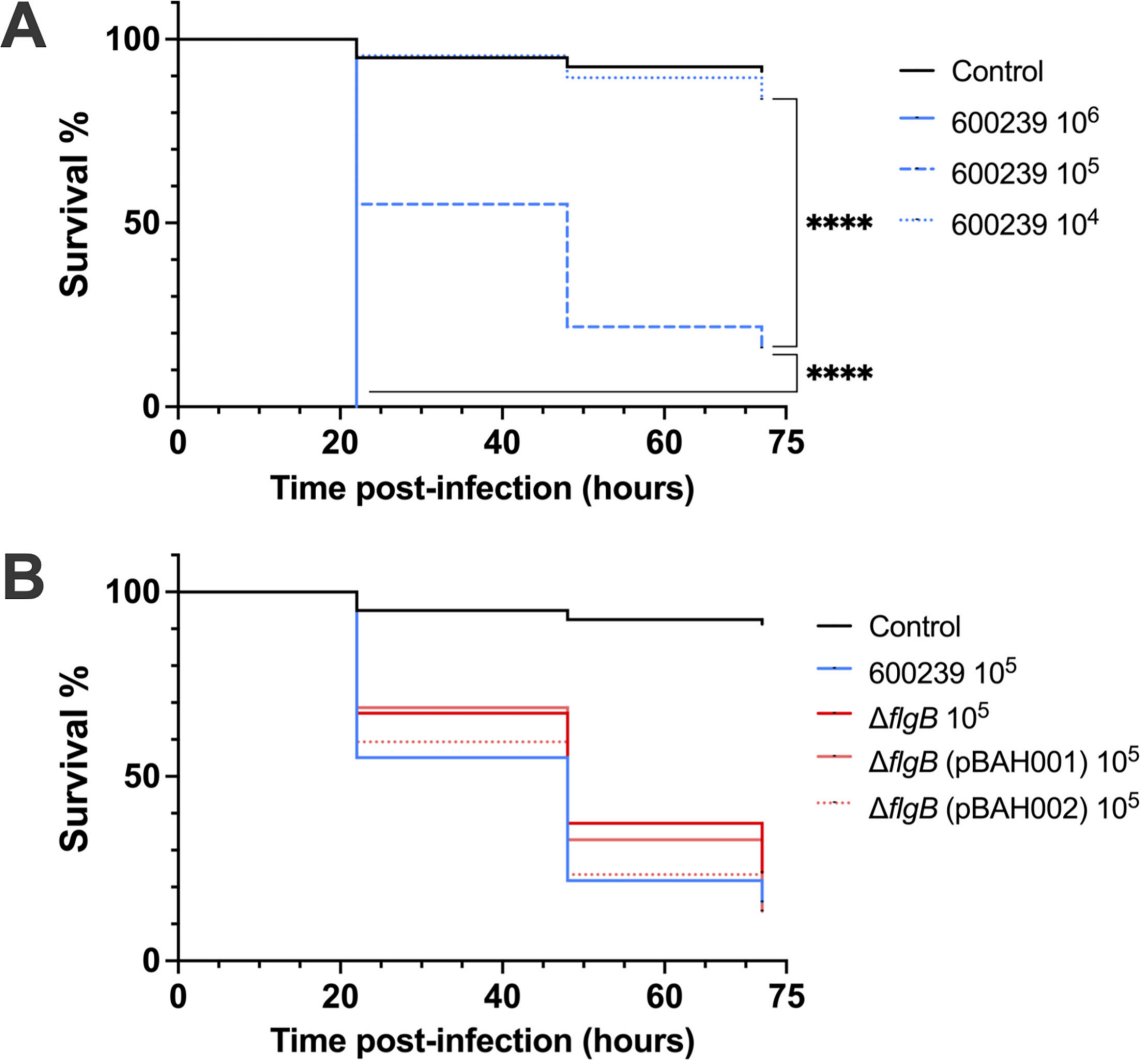
*In vivo* virulence of *A. caviae* strains in the *G. mellonella* larvae model. To assess dose response, *G. mellonella* larvae (*n* = 20–24 per group) were infected with 10^6^, 10^5^, or 10^4^ CFU of the wild-type 600239 strain (**A**). To assess virulence across strains, *G. mellonella* larvae (*n* = 20–24 per group) were infected with 10^5^ CFU of the 600239, Δ*flgB* mutant, or complemented strains, Δ*flgB* (pBAH001) or Δ*flgB* (pBAH002) (**B**). Larvae were infected via injection into the hemolymph. Controls were injected with an equal volume of inoculum diluent. Larvae were maintained at 37°C and survival was assessed at 22, 48, and 72 h post-infection. Survival curves were individually compared between each experimental group using the Log-rank (Mantel-Cox) test and data pooled from three independent experiments are shown (****, *P* ≤ 0.0001; ns, non-significant). Survival was not significantly different between any of the experimental groups infected with 10^5^ CFU.

Small animal models are often not suitable for *in vivo* assessment of enteric pathogens, including *Aeromonas,* as infection does not result in diarrhea, the main symptom of enteric infection in humans (25, 53, 54). Therefore, we assessed *in vivo* virulence in a *G. mellonella* larval model. Larvae were injected with 10^6^, 10^5^, or 10^4^ CFU of each strain and survival was monitored for 72 h. Survival between experimental groups was compared by the Log-rank (Mantel-Cox) test. All four *A. caviae* strains exhibited a dose response, and survival was significantly increased when infecting with 10^4^ CFU vs 10^5^ CFU (*P* < 0.0001), and with 10^5^ CFU vs 10^6^ CFU (*P* < 0.0001) (Fig. 6A; Fig. S3, Table S2). Infections with 10^6^ CFU yielded <2% survival rates in all experimental groups by 22 h post-infection, precluding survival comparisons at this dose (Table S2). In contrast, when infectedg with 10^4^ CFU, survival rates in all four experimental groups remained >56% and never decreased below the threshold of 50% survival (Table S2). Therefore, we assessed survival with 10^5^ CFU, which yielded an effective range of survival to measure the impact of genetic changes (Fig. 6A; Fig. S3, Table S2).

Survival was greater with Δ*flgB* compared to wild-type 600239 at each infection time point. Although not statistically significant, the overall difference in survival rates between the wild type and mutant is trending toward significance (*P* = 0.0958) (Fig. 6B; Table S2). Survival in Δ*flgB* (pBAH001) and Δ*flgB* (pBAH002) infection was not significantly different compared to wild-type or Δ*flgB* infection (Table S2).

## DISCUSSION

There is significant and expanding evidence that *A. caviae* is an independent human pathogen associated with numerous clinical pathologies (5, 6, 12, 22, 23, 28, 40). Recent studies demonstrate that while four *Aeromonas* species account for 95% of all human *Aeromonas* infections, *A. caviae* is responsible for the most significant burden (6). The prevalence of *A. caviae* compared to other *Aeromonas* species, across various infectious diseases, geographic locations, age cohorts, and socioeconomic demographics highlights *A. caviae* virulence (5, 6, 22, 23). However, historical uncertainty in identifying *A. caviae* as a human pathogen, as well as barriers to reliable speciation of *Aeromonas* precluding routine speciation of *Aeromonas* isolates in surveillance studies and clinical settings, has contributed to the lack of characterization of virulence factors specific to *A. caviae* (16, 17, 54). In the current study, we conducted comprehensive comparative genomic analyses (41, 42) in a large sample set of human *Aeromonas* isolates from GEMS (*n* = 431) (11, 22) and identified an *flgB* gene variant as a representative putative *A. caviae*-specific virulence factor. This *flgB* variant was significantly more prevalent among *A. caviae* isolates than in the other *Aeromonas* species and was predicted to encode a polar flagellar basal body protein (30, 31) putatively involved in flagellar assembly (32, 33). Notably, our phylogenetic analysis of the *flgB* gene across the 431 GEMS *Aeromonas* isolates, encompassing eight species, confirmed species specificity of *flgB*, with the exception of the *flgB* of *A. sanarellii* and *A. taiwanensis*. This data aligned with our comparative genomic analysis, wherein the *A. taiwanensis* (*n* = 4) and *A. sanarellii* (*n* = 8) isolates are 12 of the 201 (5.9%) non-*A*. *caviae Aeromonas* species isolates identified by the comparative genomic analysis to contain a *flgB* gene similar to the *A. caviae-*specific *flgB* variant. To note, it is unknown if the changes across species-specific *flgB* variants result in functional or antigenic variation. We hypothesized a putative role for *flgB* in virulence as the *A. caviae* polar flagella (37) have been demonstrated to mediate swimming motility (34–36) and host-cell adherence (36–39)*,* both of which are important tenants of virulence for enteric pathogens (8, 31, 34, 35).

Our large-scale comparative genomic analyses conducted in reliably speciated isolates are important advances in assessing *A. caviae*-specific virulence factors. Previous studies of *A. caviae* virulence factors have been limited, both in number and in scope (12, 27, 29, 55, 56). Numerous studies have characterized the virulence of *Aeromonas* isolates by screening for the presence/absence of a limited number of previously characterized virulence genes commonly present in other human-associated *Aeromonas* species isolates (12, 27, 55, 56). This approach is limited as it perpetuates a static understanding of *Aeromonas* virulence factors, minimizes the discovery of new virulence factors, and operates on the assumption that those limited virulence factors are determinants of virulence for all *Aeromonas* species. Notably, *A. caviae* lacks several common *Aeromonas* virulence factors included in the virulence screens, such as aerolysin, a T3SS, and heat-stable enterotoxin (*ast*), which resulted in historic assumptions that *A. caviae* was non-pathogenic (12, 25–29). However, the accumulating epidemiological data paint a very different picture, wherein *A. caviae* is emerging as one of the most prevalent *Aeromonas* species underlying human *Aeromonas* infections and pathologies (5, 6, 12, 23, 28, 40). Indeed, Chong et al. recently conducted an expansive analysis characterizing virulence genes in 565 *A*. *caviae* isolates, derived from both intestinal and extraintestinal human infections, as well as zoonotic and environmental sources (29). While this study is exciting and contributes to the characterization of *A. caviae* virulence genes, this work focused solely on *A. caviae* and did not include any other *Aeromonas* species in the analysis, thereby precluding the ability to assess differences in virulence gene content across species. In the current study, we applied extensive genomic comparisons, using a large sample set of relevant, reliably speciated clinical isolates, with inter-species comparisons, to systematically assess *A. caviae*-specific virulence factors and ultimately identified *flgB* as a putative *A. caviae*-specific virulence factor. *A. caviae*-specific virulence factors remain poorly characterized (27, 29), highlighting the significance of this work in developing a platform to identify and characterize virulence factors specific to *A. caviae*.

By generating an isogenic *flgB* deletion mutant and two distinct plasmid complementation constructs (Table 1), we developed a system to examine the role of a representative putative *A. caviae*-specific virulence factor, *flgB*, in aspects of bacterial virulence (motility and polar flagella production) and host–pathogen interactions (adherence, proinflammatory cytokine production, and *in vivo* virulence). We establish the essential role of *A. caviae flgB* in polar flagellar assembly and swimming motility (Fig. 1 and 2). Swimming motility and polar flagella assembly were abolished in the mutant and functionally complemented with expression from both the osmoregulated *E. coli* P*_ompC_* and the native promoter, demonstrating *flgB* is required for motility and polar flagellar assembly in *A. caviae*.

The host–pathogen interactions of *A. caviae* in human gastrointestinal infection remain understudied, and notably, it is unknown where *A. caviae* infects the human gastrointestinal tract (51). To address this, we examined the effects of *flgB* deletion on host–pathogen interactions, specifically adherence and proinflammatory cytokine production, in HT-29 and Caco2 human intestinal epithelial cell lines, representative of colonic cells and small intestinal epithelial cells, respectively (38, 52). In line with previous studies characterizing *A. caviae* adherence in human cell lines (23, 36–39, 48–50), we observed adherence of our *A. caviae* strains to both human intestinal cell lines. Interestingly, adherence of the wild-type *A. caviae* was greater in the HT-29 cells compared to Caco2 cells. While deletion of *flgB* significantly decreased adherence to HT-29 cells compared to the wild type (Fig. 3A), adherence was not rescued by complementation (Fig. 3A). In contrast, adherence to the Caco2 cells was similar across wild-type, mutant, and complemented strains (Fig. 3B). These data suggest the *A. caviae* polar flagella plays a role in mediating adherence to HT-29 cells but is not essential for adherence to Caco2 cells. This indicates other factors may be involved in adherence to these cells, in addition to the polar flagella. To characterize the host response to *A. caviae* infection, we examined proinflammatory cytokine production. We observed significant induction of IL-8, IL-13, IL-1β, and IL-6 in both HT-29 and Caco2 cell lines with wild-type 600239 infection (Fig. 4A through E and 5A through E); IFN-γ was significantly induced with wild-type infection in the HT-29 cells only (Fig. 4C). These data corroborate a previous study documenting IL-8 production in *in vitro* infection with clinical *A. caviae* isolates (23). Additionally, production of IL-8, IL-1β, and IL-6 is known to be induced by TLR5 recognition of flagellin (8, 57), the polar flagella structural subunit, and IFN-γ has been reported to modulate an inflammatory response to flagellin in the gut (58). As functional polar flagella are not assembled in our *flgB* mutant (Fig. 2), we anticipate a lack of surface-exposed flagellin in the *flgB* mutant. Our findings align with this as *flgB* deletion significantly decreased production of IL-8, IL-13, IL-1β, and IL-6 in both cell lines, and of IFN-γ in the HT-29 cells. Overall, these results demonstrate that the *A. caviae* polar flagella are involved with eliciting a proinflammatory response to *A. caviae* in both human intestinal cell lines. Additionally, the cumulative differences observed in adherence and cytokine production between the HT-29 and Caco2 cell lines could indicate possible “tropism” of *A. caviae* within the gastrointestinal tract. This tropism will need to be examined in human intestinal enteroid models, which are developed from human intestinal biopsies from various sections of the gastrointestinal tract. Therefore, these models contain greater cellular complexity than immortalized cell lines and are representative of the colon and various sections of the small intestine (59).

*In vivo* models are critical for investigating host–pathogen interactions of human pathogens. However, *in vivo* assessment of enteric pathogens is significantly limited due to a lack of relevant small animal models, as diarrhea, the hallmark phenotype of human enteric infections, is often not produced in these models (25, 53, 54). Mouse models can be utilized as a survival-based virulence screening platform; however, in limited studies, infection with human diarrheal *A. caviae* isolates yielded LD_50_ values of 2–6 × 10^9^ CFU (25, 54). The *G. mellonella* larval model is a survival-based virulence screening platform that lacks the inherent hurdles of murine models (53, 60), thereby facilitating cost-effective interrogation of greater numbers of organisms, translating to more powered assessments, without the need for regulatory approval, skilled technical training, or specialized personnel and facilities. This model has been used to assess the virulence of other enteric pathogens, *Shigella* spp., *C. jejuni*, and *Y. enterocolitica,* thus confirming this model as a valuable screening platform for enteric pathogens (53, 60). The *G. mellonella* model was previously utilized in a single study to assess the virulence of environmental *A. hydrophila, veronii,* and *salmonicida* isolates. However, our current study will be the first application of this model for assessing the virulence of *A. caviae,* and of a human-associated clinical *Aeromonas* isolate (61). We achieved consistent and reproducible dose responses for all *A. caviae* strains, demonstrating the suitability of the model to assess *A. caviae* virulence *in vivo* (Fig. 6 and Table S2). Deletion of *flgB* modestly, but not significantly, increased survival compared to the wild type. This data suggests *A. caviae* pathogenesis in *G. mellonella* most likely involves additional factors.

In conclusion, we leveraged the power of comparative genomics and a large sample set of accurately speciated human clinical *Aeromonas* isolates (*n* = 431) to identify genetic factors specific to *A. caviae* with putative roles in virulence. We characterized one representative putative *A. caviae-*specific virulence factor, *flgB*, and demonstrated its essential role in aqueous motility and polar flagellar formation using a deletion mutant and complementation constructs generated in this study. Deletion of *flgB* significantly decreased bacterial adherence to HT-29 cells and interrupted the production of proinflammatory cytokines in both HT-29 and Caco2 human intestinal cells. Lastly, we characterized *in vivo* virulence of *A. caviae* in the *G. mellonella* larval survival model for the first time and observed a non-significant increase in survival with *flgB* deletion compared to the wild type in this model. Taken together, these data suggest a role for *flgB* in bacterial virulence and host–pathogen interactions. Lastly, we established a framework for the identification and characterization of *A. caviae*-specific virulence factors, coupling comparative genomic analyses with the genetic systems, functional assays, and the *in vivo* and *in vitro* models we optimized here.

## MATERIALS AND METHODS

### Bacterial strains, culture conditions, and reagents

Bacterial strains utilized in this study are listed in Table 1. Strains were cultured from frozen stocks in Mueller-Hinton medium (BD) [for 600239, Δ*flgB,* and Δ*flgB* (pBAH002)] or Lysogeny broth (LB) medium (MP Biomedicals) [for Δ*flgB* (pBAH001)] and incubated overnight at 37°C unless otherwise stated. For antibiotic selection, 50 μg/mL neomycin (Neo50) (Sigma) or 20 μg/mL chloramphenicol (Cm20) (Sigma) was added to culture media when applicable. The Δ*flgB* (pBAH001) strain was cultured in LB containing 3 g/L NaCl or 10 g/L NaCl, as expression from the *E. coli ompC* promoter (P*_ompC_*) can be increased by increasing the osmolarity of culture conditions (45, 46). However, the regulation of P*_ompC_* is not significantly stringent enough to eliminate gene expression. Molecular biology techniques were performed according to standard protocols (62). GoTaq Green DNA polymerase master mix (Promega) or PfuUltra II Fusion HS DNA Polymerase (Agilent) was utilized for PCR amplifications. Restriction enzymes and T4 DNA ligase were acquired from NEBioLabs. Primers were generated by Integrated DNA Technologies. All primers and PCR amplification conditions are listed in Table S1. Plasmids and plasmid constructs are listed in Table 1.

### Comparative genomics

For all comparative genomic analyses, we utilized draft genomes we previously generated for a representative set of 431 clinical human-associated *Aeromonas* isolates collected in the GEMS (4, 11, 22). Sequencing, genome assembly, and phylogenetic comparison were conducted as previously described (22, 63). Eight species are represented in this sample set as determined by our prior phylogenetic comparison analysis: *A. caviae* (*n* = 230)*, A. veronii* (*n* = 124)*, A. enteropelogenes* (*n* = 29), *A. dhakensis* (*n* = 21), *A. jandaei* (*n* = 14), *A. taiwanensis* (*n* = 8), *A. sanarellii* (*n* = 4), and *A. hydrophila* (*n* = 1). Assembled genomes were annotated with Prokka (version 1.14.6) (64), and the Generated General Feature Formats were analyzed using Roary (version 3.13.0) (41). *A. caviae* genomes (*n* = 230) were compared to the genomes of the other seven *Aeromonas* species identified in GEMS (*n* = 201) using Scoary (version 1.6.16) (42). Results were evaluated with Bonferroni’s method for multiple comparisons within Scoary analysis (42). To identify *A. caviae-*specific putative virulence factors, we first categorized *A. caviae-*specific genes as those significantly more abundant in *A. caviae* isolates than in the isolates of the other seven species (*P* < 0.0005), and present in ≥90% of *A. caviae* isolates and in ≤10% of other *Aeromonas* species isolates. We manually assessed the *A. caviae*-specific genes and identified putative virulence factors as genes predicted to encode virulence factors in other enteric pathogens, such as genes involved in bacterial motility and adherence (8, 31, 34, 35). An *A. caviae*-specific *flgB* gene variant, predicted to encode a polar flagella basal body rod protein (30, 31), was selected as a representative putative virulence factor, as polar flagella can be assessed with well-defined functional assays.

### Phylogenetic analysis of *flgB*

BLASTN (65) and MUSCLE (66) were used to align the *flgB* nucleotide sequence across the 431 *Aeromonas* isolates to assess divergence. IQ-TREE (v2) was utilized to infer a midpoint-rooted maximum-likelihood phylogeny (67). The inferred phylogeny was visualized with FigTree (v 1.4.4) (https://tree.bio.ed.ac.uk/software/figtree/). Nucleotide sequences of *flgB* across the 431 *Aeromonas* isolates were translated (68), and BLASTP (65) was utilized to assess amino acid identity and similarity. Nucleotide and amino acid sequences of representative species-specific *flgB* variants were aligned using CLUSTALW (version 2.1) (69) using default parameters to generate the alignments for Fig. S1.

### Lambda Red mutagenesis

Stable chromosomal *flgB* deletion was carried out in a single, representative clinical GEMS *A. caviae* isolate 600239 utilizing the Lambda Red recombineering system as previously described (43, 44). Briefly, plasmid pKM200 (44) was introduced into the 600239 strain by electroporation; transformants were selected on LB+Cm20 at 30°C and screened for the pKM200 plasmid via PCR using primer set JMM01-F/R (Table S1). Expression of the Lambda Red recombinase was induced with 1 mM IPTG, then 600239 was heat-shocked at 42°C for 15 min and made electrocompetent (44).

Using primer set BAH01-F/R (Table S1), 66–67 bp of the sequence flanking *flgB* were PCR amplified onto a kanamycin/neomycin resistance cassette (*aph*) from helper plasmid, pKD4 (43). The ~1.6 kb product was electroporated into 600239 expressing the Lambda Red recombinase, plated on MH+Neo50, and neomycin-resistant isolates were screened by PCR for the Δ*flgB::aph* mutation using primer set BAH02-F/R (Table S1). The *aph* cassette was removed from the genome at FRT sites by an FLP flippase carried on the pCP20 plasmid (43). pCP20 was introduced into 600239 Δ*flgB* by electroporation and selected on LB+Cm20 at 30°C. Flippase activity was induced by incubation at 42°C, and transformants were screened for loss of phenotypic resistance (43) by patching on MH and counterselection on MH+Neo50. Loss of the *aph* cassette was verified by PCR in resultant clones using primer set BAH02-F/R (Table S1). Whole-genome sequencing (SeqCenter) was utilized to confirm *flgB* deletion, removal of the *aph* cassette, and absence of other mutations/genetic effects of the molecular engineering.

### Generation of expression constructs for complementation

Plasmid expression constructs for *trans* complementation of *flgB* were generated in pSEC10 (45, 46) for *flgB* expression from the osmoregulated *E. coli ompC* promoter (P*_ompC_*) (pBAH001) and pSEC10-M (47) for expression from the native promoter (pBAH002) (Table 1). To generate pBAH001, *flgB* was PCR amplified from 600239, with 5′ BamHI and 3′ NheI restriction sites, using PfuUltra II Fusion HS DNA Polymerase (Agilent), CleanAmp 7-deaza-dGTP Mix (TriLink), and primer set BAH03-F/R (Table S1). The resulting 420 bp product was purified by phenol-chloroform extraction, precipitated with 125 mM NaCl and ethanol, rescued in the pCR-Blunt II-TOPO vector (Invitrogen) using the Zero Blunt TOPO PCR Cloning Kit (Invitrogen), transformed into One Shot TOP10 Chemically Competent *E. coli* (Invitrogen) per manufacturer’s instructions, and selected on MH+Neo50. The resulting construct, pCR-Blunt II-TOPO::*flgB* (Table 1), was confirmed by restriction enzyme digest and Sanger Sequencing (GENEWIZ). Plasmid pSEC10 (45, 46) was digested with BamHI and NheI, and a ~6.3 kb fragment was excised following agarose gel electrophoresis and gel purified (Invitrogen PureLink Quick Gel Extraction and PCR Purification Combo Kit). pCR-Blunt II-TOPO::*flgB* was digested with BamHI and NheI, separated by agarose gel electrophoresis, and a 420 bp fragment was excised, electroeluted, and purified by phenol-chloroform extraction. The purified 420 bp fragment was ligated into the BamHI/NheI site of pSEC10, downstream of P*_ompC_*, transformed into TOP10 *E. coli* as above*,* selected on MH+Neo50, and PCR-screened using primer set BAH03-F/R (Table S1), yielding pBAH001 (Table 1).

To generate pBAH002, *flgB*, along with 500 bp of upstream sequence, was PCR amplified from 600239, with 5′ NheI and 3′ AvrII restriction sites, using PfuUltra II Fusion HS DNA Polymerase (Agilent), CleanAmp 7-deaza-dGTP Mix (TriLink), and primer set BAH04-F/R (Table S1). The 920 bp product was purified by phenol-chloroform extraction, precipitated with 125 mM NaCl and ethanol, rescued in pCR-Blunt II-TOPO (Invitrogen), transformed into TOP10 *E. coli* as above, and selected on MH+Neo50. The resulting construct, pCR-Blunt II-TOPO::*flgB*+500 (Table 1), was confirmed by restriction enzyme digest and Sanger Sequencing (GENEWIZ). pCR-Blunt II-TOPO::*flgB*+500 was digested with NheI and AvrII, and a 920 bp fragment was electroeluted from an agarose gel fragment, purified by phenol-chloroform extraction, ligated into the NheI/AvrII site of plasmid pSEC10-M (47), transformed into TOP10 *E. coli* as above, selected on MH+Neo50, and PCR-screened using primer set BAH04-F/R (Table S1), yielding pBAH002 (Table 1). Purified pBAH001 and pBAH002 were separately electroporated into the Δ*flgB* 600239 strain, selected on MH+Neo50, and PCR-screened using primer sets BAH03-F/R and BAH04-F/R, respectively (Table S1). Introduction of pBAH001 and pBAH002 and the host genome sequence was confirmed by whole-genome sequencing (SeqCenter).

### Growth curve assays

Overnight broth cultures of each strain (Table 1) were diluted 1:500 in fresh media and assayed in technical triplicate in 96-well, flat-bottom plates at 37°C with agitation. The OD_600_ was measured every 3 min for ~16.5 h using the Stratus (MRODX1r2) continuous plate reader (Cerillo). Fresh culture media served as baseline controls. Results were collated using the Cerillo Labrador software (v2.3.3). Two independent experiments were conducted.

### Motility assays

Overnight broth cultures of each strain (Table 1) were inoculated into the center of soft agar plates (0.25% agar) with a sterile toothpick. Plates were incubated at 37°C (not inverted) and migration from the inoculation point (diameter) was measured ~22 h post-inoculation. Three independent experiments were performed.

### Transmission electron microscopy

For all strains (Table 1), overnight cultures on agar plates were submitted to the University of Maryland, Baltimore Electron Microscopy Core for negative staining and transmission electron microscopy imaging as described previously (70). Single colonies were transferred to glow-discharged, formvar carbon-stabilized grids by floating the grid over a single colony in a droplet of filtered water to retain bacterial conformation and morphology. Grids were stained with 2% aqueous phosphotungstic acid, air-dried, and imaged in an FEI Tecnai T12 (Thermo Fisher) transmission electron microscope at 80 kV with an AMT bottom-mount camera.

### Cell culture and adherence assays

HT-29 (ATCC HTB-38) and Caco2 (ATCC HTB-37) cell lines were cultured in 75 cm^2^ flasks at 37°C, 5% CO_2_ in Dulbecco’s Modified Eagle Medium (DMEM) (Gibco) supplemented with 10% and 20% heat-inactivated fetal bovine serum (FBS) (Gibco), respectively.

For adherence assays, cells were seeded into 12-well plates (Corning) and cultured to confluence. To prepare the inocula, overnight broth cultures of each strain (Table 1) were diluted in fresh media, cultured to 0.5 OD_600_, pelleted, and resuspended in an equal volume of DMEM(-FBS) (Gibco). Inocula were diluted to MOI = 0.05 in DMEM(-FBS). Inocula were serially diluted and plated for enumeration of CFU.

Confluent monolayers of HT-29 or Caco2 cells were infected with each strain in technical triplicate, at MOI = 0.05 for 4 h (37°C, 5% CO_2_). DMEM(-FBS) served as a negative control. Four hours post-infection, spent media supernatants were collected for cytokine production assays, then monolayers were washed three times with fresh DMEM(-FBS), lysed with Triton-X (ThermoFisher), serially diluted, and plated for enumeration of CFU of adhered bacteria. Three independent experiments were performed for both cell types.

### Cytokine assays

Spent media supernatants from the adherence assays in HT-29 and Caco2 cells were collected 4 h post-infection, centrifuged (12,000 × *g*, 5 min, 4°C) to remove bacterial/cellular debris, and stored at −80°C until use. MSD U-PLEX Biomarker Group 1 (human) Multiplex Assays (K15049K-1) (Mesoscale Diagnostics [MSD]) were utilized to assess the production of 10 pro-inflammatory cytokines: interferon-gamma (IFN-γ), tumor necrosis factor-alpha (TNF-α), and interleukins IL-1β, IL-2, IL-4, IL-6, IL-8, IL-10, IL-12p70, and IL-13. Each sample was run in technical duplicate, and assays were conducted per the manufacturer’s instructions. Data were analyzed using the MSD Discovery Workbench (version 4.0; https://www.mesoscale.com/en/products_and_services/software).

### *G. mellonella* survival assays

Larvae of the Greater Wax Moth, *G. mellonella,* were utilized as an alternative model for screening virulence *in vivo* as described previously (53, 71), with minor modifications. Briefly, *G. mellonella* larvae (Vanderhorst Wholesale; https://www.waxworms.net) weighing 210–300 mg, and showing no signs of disease (active, cream-colored), were sorted into Petri dishes with sawdust bedding one day prior to infection (*n* = 20–24 larvae per group); larvae were utilized within 2–3 days of receipt. Prior to injection, larvae were immobilized at 4°C (30–40 min), then incubated on ice immediately preceding injection. A group of non-injected larvae served as environmental controls.

For inocula preparation, overnight broth cultures of each strain (Table 1) were diluted 1:100 in fresh media, cultured to 0.5 OD_600_, pelleted, and resuspended in an equal volume of DMEM (Gibco). To assess dose response, inocula were diluted to 10^8^, 10^7^, or 10^6^ CFU/1 mL DMEM. Ten microliters of inoculum were injected into the hemolymph via injection into the last, right proleg using 1 cc insulin syringes (31G needle, 5/16) (BD). This translated to 10^6^, 10^5^, or 10^4^ CFU in the larvae, respectively. Controls were injected with an equal volume of inocula diluent, DMEM. Larvae were incubated at 37°C and survival was assessed at 22, 48, and 72 h post-infection. Larvae were deemed dead with failure to move upon prodding. Inocula were serially diluted and plated for enumeration of CFU. Three independent experiments were performed.

### Statistics

GraphPad Prism (Version 10.4.1) was utilized to visualize data and conduct analysis for all statistical assessments, with the exception of those conducted within the Scoary pipeline (42). Data for motility, adherence, and cytokine production assays were assessed by ordinary one-way ANOVA with Tukey’s test for multiple comparisons; data are shown as mean ± standard deviation. Survival in the *G. mellonella* model was plotted in Kaplan-Meier curves, and the log-rank (Mantel-Cox) test was used to compare survival between two groups across all comparisons. Significance is indicated as follows: ****, ***, **, and *, *P* ≤ 0.0001, *P* ≤ 0.001, *P* ≤ 0.01, and *P* ≤ 0.05, respectively.

## Data Availability

All *Aeromonas* genomes utilized in this study have been released and are available in BioProject PRJNA607550 and PRJNA1252136.

## SUPPLEMENTAL MATERIAL

Supplemental tables and figures jb.00339-25-s0001.pdf Tables S1 and S2, and Figures S1 and S2.

ASM does not own the copyrights to Supplemental Material that may be linked to, or accessed through, an article. The authors have granted ASM a non-exclusive, world-wide license to publish the Supplemental Material files. Please contact the corresponding author directly for reuse.
