# Supplementary material for: Characterization of the role of putative Aeromonas caviae-specific virulence factor, flgB, in virulence and host–pathogen interactions
Source: J Bacteriol. 2025 Dec 29;208(1):e00339-25. doi: 10.1128/jb.00339-25 (PMC12826063; doi:10.1128/jb.00339-25)
Supplement: Supplemental tables and figures — Tables S1 and S2, and Figures S1 and S2. [file jb.00339-25-s0001.pdf]

SUPPLEMENTARY TABLES AND FIGURES

Supplementary Table 1. Primers used in this study

Supplementary Table 2. Survival comparisons in *Galleria mellonella* larvae infected with *A. caviae* strains

Figure S1. Phylogenetic analysis and comparative data of the *flgB* gene across human-associated clinical *Aeromonas* isolates. The *flgB* gene was examined across a representative set of *Aeromonas* isolates (n=431) from the Global Enteric Multicenter Study: *A. caviae* (n=230), *A. veronii* (n=124), *A. enteropelogenes* (n=29), *A. dhakensis* (n=21), *A. jandaei* (n=14), *A. taiwanensis* (n=8), *A. sanarellii* (n=4), and *A. hydrophila* (n=1). The *flgB* nucleotide and amino acid sequences were aligned to the *flgB* sequence of a representative GEMS isolate and allelic variation, amino acid identity, and amino acid similarity are presented in the table in Panel B. Species-specific clades are marked by color in the mid-point rooted maximum-likelihood phylogeny (Panel A). Representative species-specific *flgB* nucleotide and amino acid sequences were aligned and are presented in Panel B and C.

Figure S2. Growth curves for *A. caviae* strains. Overnight broth cultures of the 600239 wild-type, Δ*flgB* mutant, and complemented strains, Δ*flgB* (pBAH001) (*flgB* expression from the osmoregulated *E. coli ompC* promoter (P_{ompC})) and Δ*flgB* (pBAH002) (expression from the native promoter) were diluted in fresh media (in technical triplicate) and OD_{600} measurements were taken every 3 minutes for ~16.5 hours using the Cerillo Stratus (MRODX1r2) continuous plate reader. Fresh media served as baseline controls. To assess fitness when modulating expression from P_{ompC}, the Δ*flgB* (pBAH001) strain was cultured in Lysogeny broth containing 3g/L NaCl (annotated P_{ompC} low) or 10g/L NaCl (annotated P_{ompC} high). Two independent experiments were performed and representative data is presented.

Figure S3. Dose response of *A. caviae* strains infecting the *Galleria mellonella* larvae model. *G. mellonella* larvae (n=20-24 per group) were infected with 10^6, 10^5, or 10^4 CFU of the Δ*flgB* mutant (Panel A) or complemented strains, Δ*flgB* (pBAH001) (Panel B) and Δ*flgB* (pBAH002) (Panel C). Larvae were infected via injection into the hemolymph; controls were injected with

equal volume of inoculum diluent. Larvae were maintained at 37°C and survival was assessed at 22, 48, and 72 hours post-infection. Survival curves were individually compared between each experimental group using the Log-rank (Mantel-Cox) test. Data pooled from three independent experiments is presented in Kaplan Meier survival curves (****, $P \leq 0.0001$).

Table S1 Primers used in this study

Primers		
**Primer**	**Description**	**Sequence (5'-3')**
JMM01-F	pKM200 confirmation primer F (M13R)	CAGGAAACAGCTATGAC
JMM01-R	pKM200 confirmation primer R (pKD46R)	CGTACTGGTTGGCAACGATC
BAH01-F	pKD4-600239 *flgB* homology region F	TAACCCCGCTCAATACCCTTTTGGCACACCTCTTGCTAAATATTCATGAAGCAGGAGGTGTGCTATG<br>GTGTAGGCTGGAGCTGCTTC
BAH01-R	pKD4-600239 *flgB* homology region R	TGTTGAGGCGAACGGACTGCGCGCTCATGGCAGAGCCTGCAATGTCAAAGACCTTGAACAAACTCA<br>GGTCCATATGAATATCCTCCTTAGTTCC
BAH02-F	Sequence flanking 600239 *flgB* homology region F	GAGCTACAGCTTGCTCGGC
BAH02-R	Sequence flanking 600239 *flgB* homology region R	GAGGCCTGCTTGTCGGAC
BAH03-F	600239 *flgB* (5' BamHI, 3' NheI) pBAH001 insert F	AAAGGATCCATGGCAATTTCATTCGACAAGGC
BAH03-R	600239 *flgB* (5' BamHI, 3' NheI) pBAH001 insert R	AAAGCTAGCTCATCCTTGCTCTCCTTTCAGTG
BAH04-F	600239 *flgB* +500bp upstream (5' NheI, 3' AvrII) pBAH002 insert F	AAAGCTAGCGCGGCCGGGTACCTTG
BAH04-R	600239 *flgB* +500bp upstream (5' NheI, 3' AvrII) pBAH002 insert R	AAACCTAGGTCATCCTTGCTCTCCTTTCAGTG

PCR Amplification Conditions			
**Primer Set**	**Amplification Conditions**	**Cycles**	**DNA Polymerase**
JMM01-F/R	93°C (30s), 52°C (20s), 72°C (1 minute)	30 cycles; initial denaturation at 94°C (1 minute); final extension at 72°C (2 minutes)	GoTaq (Promega)
BAH01-F/R	*step 1:* 95°C (30s), 51°C (20s), 72°C (1.75 minutes)<br>*step 2:* 95°C (30s), 56°C (20s), 72°C (1.75 minutes)	1 cycle followed by 29 cycles BAH01-F/R step 2; initial denaturation at 95°C (2 minutes)<br>29 cycles; final extension at 72°C (5 minutes)	GoTaq (Promega)
BAH02-F/R	93°C (30s), 54°C (20s), 72°C (2 minutes)	30 cycles; initial denaturation at 94°C (1 minute); final extension at 72°C (2 minutes)	GoTaq (Promega)
BAH03-F/R	*step 1:* 95°C (20s), 54°C (20s), 72°C (20s)<br>*step 2:* 95°C (20s), 59°C (20s), 72°C (20s)	2 cycles followed by 29 cycles BAH03-F/R step 2; initial denaturation at 95°C (2 minutes)<br>29 cycles; final extension at 72°C (3 minutes)	HS DNA Polymerase (Agilent)
BAH04-F/R	*step 1:* 95°C (20s), 54°C (20s), 72°C (20s)<br>*step 2:* 95°C (20s), 59°C (20s), 72°C (20s)	2 cycles followed by 29 cycles BAH04-F/R step 2; initial denaturation at 95°C (2 minutes)<br>29 cycles; final extension at 72°C (3 minutes)	HS DNA Polymerase (Agilent)

37

Table S2 Survival comparisons in *Galleria mellonella* larvae infected with *A. caviae* strains

Group	n (larvae)	Survival (%)			Significance (p-value)*	
		22h	48h	72h	vs 600239	vs Δ*flgB*
1 x 10^6 CFU						
Control	80	95.0	92.5	91.3	**** (<0.0001)	**** (<0.0001)
Wild-type 600239	66	0.0	0.0	0.0	N/A	ns (>0.9999)
Δ*flgB*	66	0.0	0.0	0.0	ns (>0.9999)	N/A
Δ*flgB* (pBAH001)	69	0.0	0.0	0.0	ns (>0.9999)	ns (>0.9999)
Δ*flgB* (pBAH002)	66	1.5	1.5	1.5	ns (0.3173)	ns (0.3173)
1 x 10^5 CFU						
Control	80	95.0	92.5	91.3	**** (<0.0001)	**** (<0.0001)
Wild-type 600239	69	55.1	21.7	15.9	N/A	ns (0.0958)
Δ*flgB*	67	67.2	37.3	23.9	ns (0.0958)	N/A
Δ*flgB* (pBAH001)	67	68.7	32.8	13.4	ns (0.3798)	ns (0.3233)
Δ*flgB* (pBAH002)	64	59.4	23.4	14.1	ns (0.9104)	ns (0.1086)
1 x 10^4 CFU						
Control	80	95.0	92.5	91.3	ns (0.1726)	ns (0.7597)
Wild-type 600239	67	95.5	89.6	83.6	N/A	ns (0.1083)
Δ*flgB*	66	100.0	97.0	92.4	ns (0.1083)	N/A
Δ*flgB* (pBAH001)	70	92.9	74.3	71.4	ns (0.0856)	** (0.0012)
Δ*flgB* (pBAH002)	67	83.6	70.1	56.7	*** (0.0006)	**** (<0.0001)

*determined by Log-rank (Mantel-Cox) test for comparison of survival curves

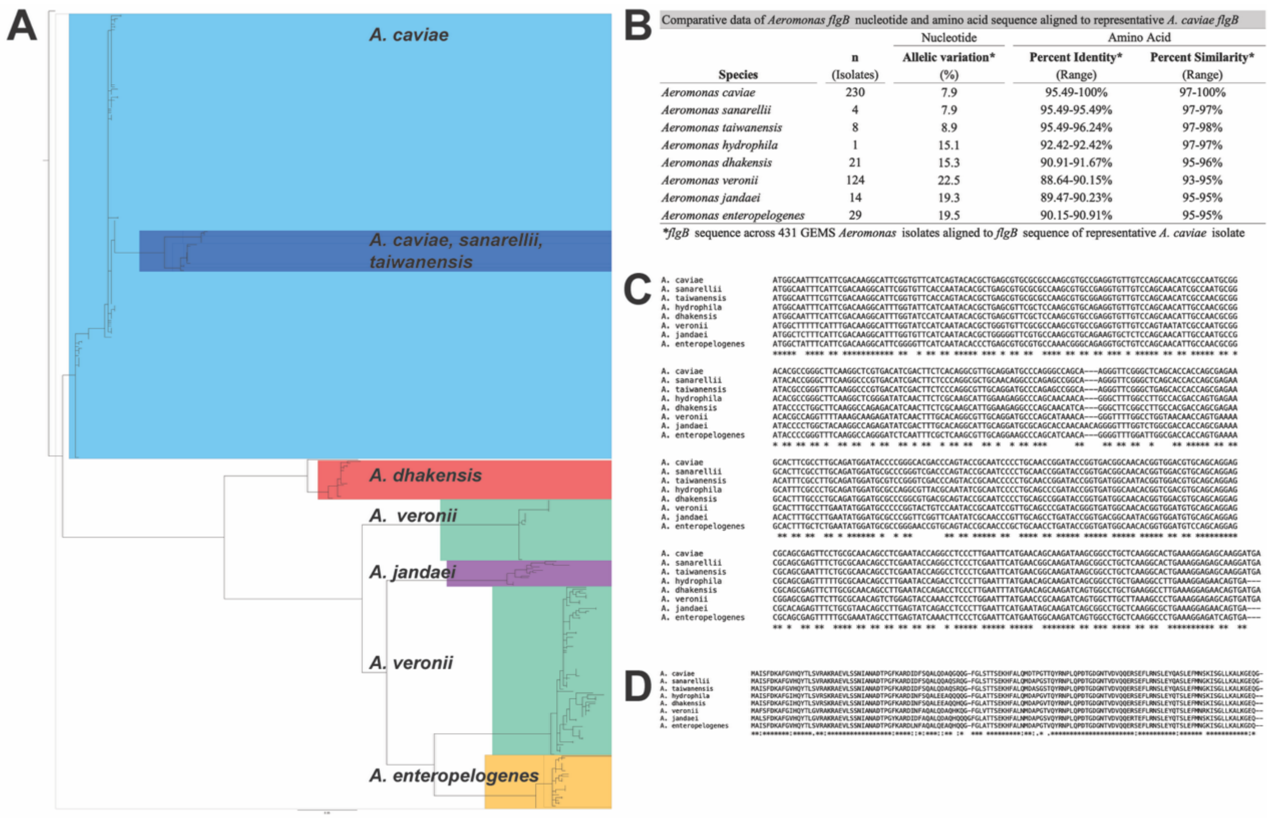

FIG S1 Phylogenetic analysis and comparative data of the *flgB* gene across human-associated clinical *Aeromonas* isolates.

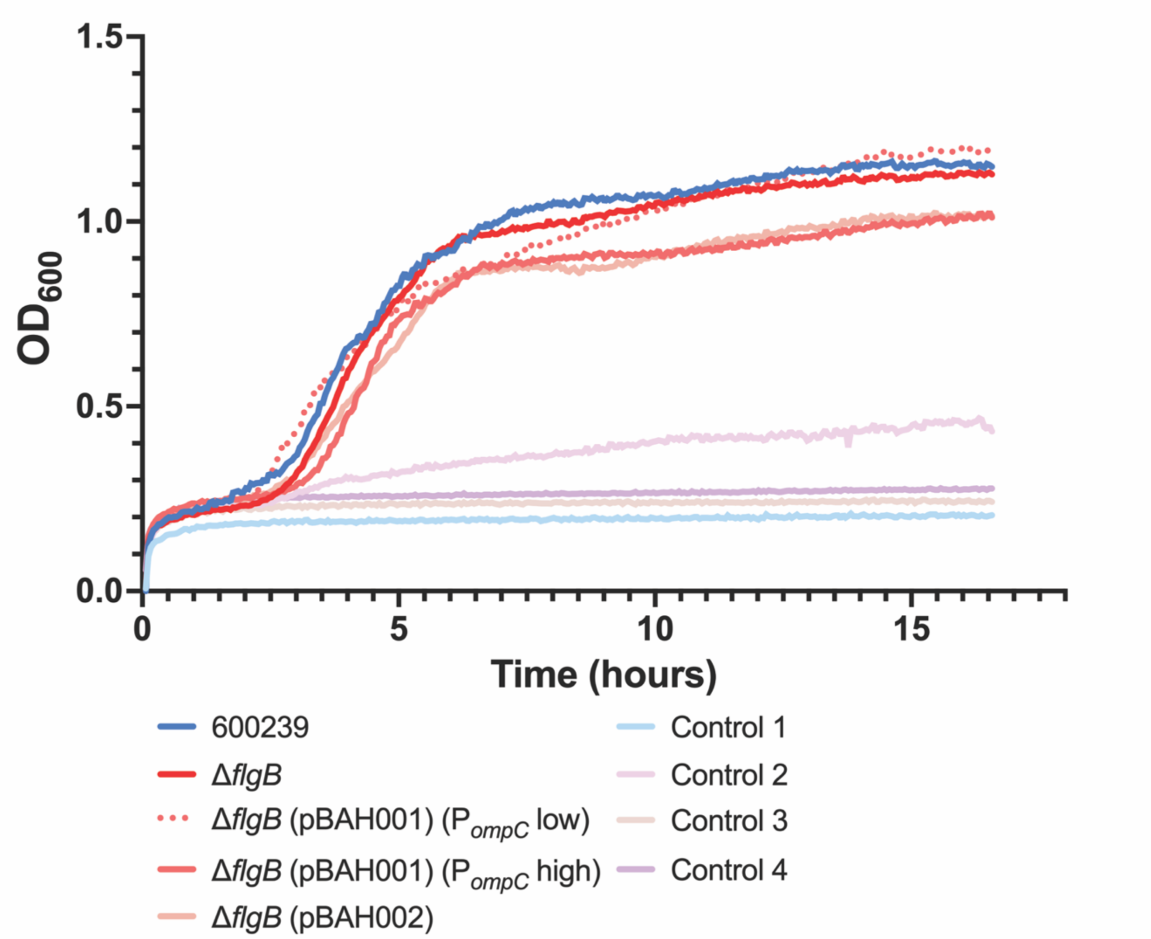

FIG S2 Growth curves for *A. caviae* strains.

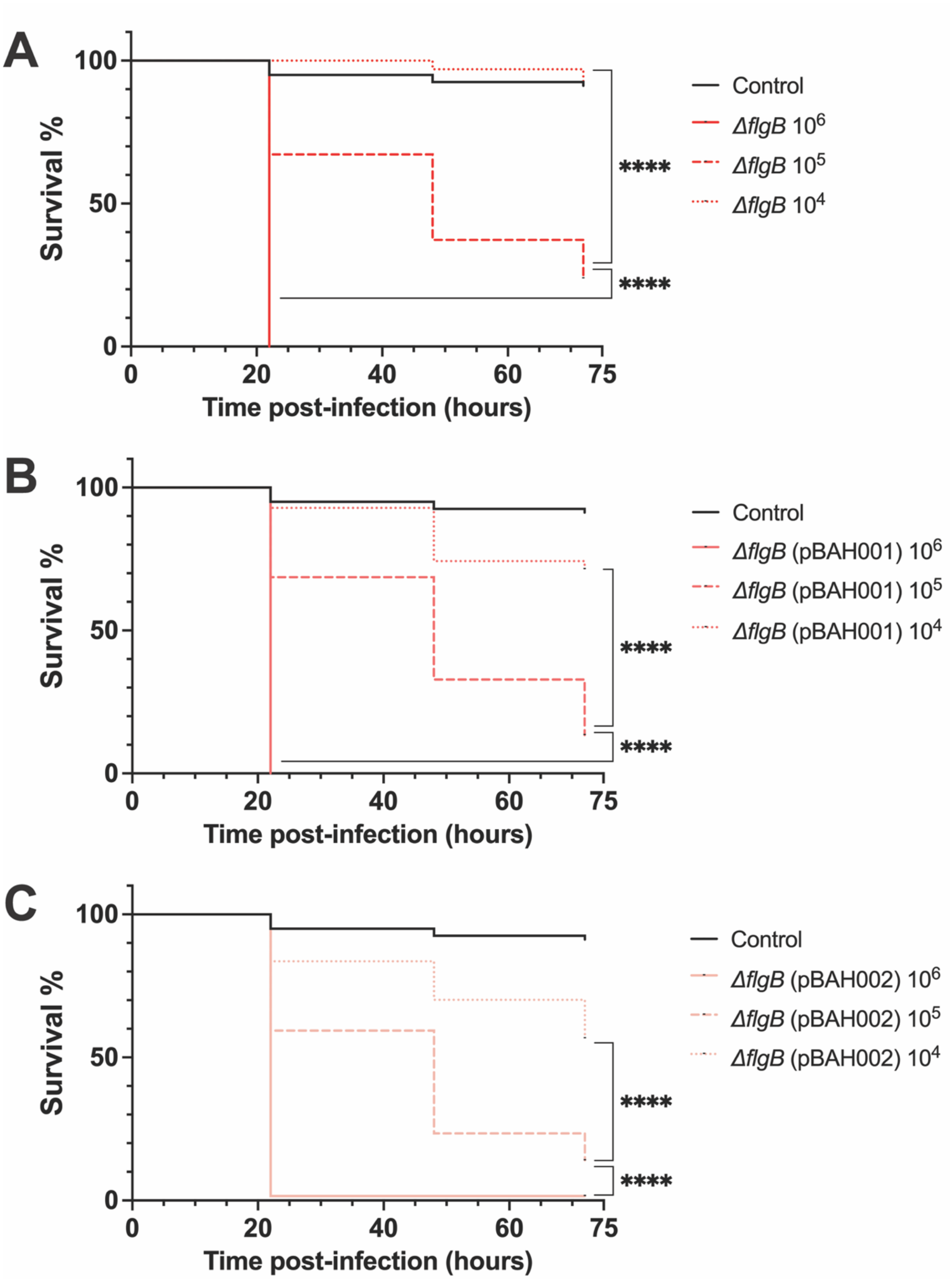

FIG S3 Dose response of *A. caviae* strains in the *Galleria mellonella* larvae model.
